# Predictors of Barefoot Plantar Pressure during Walking in Patients with Diabetes, Peripheral Neuropathy and a History of Ulceration

**DOI:** 10.1371/journal.pone.0117443

**Published:** 2015-02-03

**Authors:** Ruth Barn, Roelof Waaijman, Frans Nollet, James Woodburn, Sicco A. Bus

**Affiliations:** 1 Department of Rehabilitation, Academic Medical Centre, University of Amsterdam, Amsterdam, the Netherlands; 2 Institute for Applied Health Research, School of Health and Life Sciences, Glasgow Caledonian University, Cowcaddens Road, Glasgow, United Kingdom; Harvard Medical School, UNITED STATES

## Abstract

**Objective:**

Elevated dynamic plantar foot pressures significantly increase the risk of foot ulceration in diabetes mellitus. The aim was to determine which factors predict plantar pressures in a population of diabetic patients who are at high-risk of foot ulceration.

**Methods:**

Patients with diabetes, peripheral neuropathy and a history of ulceration were eligible for inclusion in this cross sectional study. Demographic data, foot structure and function, and disease-related factors were recorded and used as potential predictor variables in the analyses. Barefoot peak pressures during walking were calculated for the heel, midfoot, forefoot, lesser toes, and hallux regions. Potential predictors were investigated using multivariate linear regression analyses. 167 participants with mean age of 63 years contributed 329 feet to the analyses.

**Results:**

The regression models were able to predict between 6% (heel) and 41% (midfoot) of the variation in peak plantar pressures. The largest contributing factor in the heel model was glycosylated haemoglobin concentration, in the midfoot Charcot deformity, in the forefoot prominent metatarsal heads, in the lesser toes hammer toe deformity and in the hallux previous ulceration. Variables with local effects (e.g. foot deformity) were stronger predictors of plantar pressure than global features (e.g. body mass, age, gender, or diabetes duration).

**Conclusion:**

The presence of local deformity was the largest contributing factor to barefoot dynamic plantar pressure in high-risk diabetic patients and should therefore be adequately managed to reduce plantar pressure and ulcer risk. However, a significant amount of variance is unexplained by the models, which advocates the quantitative measurement of plantar pressures in the clinical risk assessment of the patient.

## Introduction

In patients with diabetes mellitus, foot ulceration as a complication of the disease is associated with significant burden and increased mortality [1]. Elevated plantar pressures during locomotion are known to contribute to the development of diabetic foot ulcers [2–9]. After healing of a foot ulcer, many patients experience ulcer recurrence, and emerging evidence suggests that elevated plantar pressures are also a significant determinant of foot ulcer recurrence [10]. It is therefore recommended that interventions should routinely include targeting of abnormal pressures [11]. But despite the significance of the role of high plantar pressure in ulcer development, its determinants are not well understood in diabetes and we are therefore currently poor at explaining which patients have or will develop increased plantar pressures.

A number of studies have investigated the relationship between clinical and structural variables and either in-shoe or barefoot plantar pressure in diverse diabetes populations demonstrating contrasting results [12–19]. Important factors include presence of foot deformity [12,14,15,18,19], limited joint mobility at the ankle and metatarso-phalangeal joints [13,16], variables related to the presence of peripheral neuropathy [13,17], presence of callus [12,18] and soft tissue thickness [20]. There is controversy surrounding the contribution of body mass as it was reported to have limited predictive value in terms of plantar pressure in subjects with diabetes in one study [14] but an important contribution in another study [12]. It is difficult to directly compare the findings from studies in this area due to the varied methodologies employed. To date, none of the studies in diabetic populations have included prediction of pressures in the midfoot region with the majority focusing on plantar pressure in the forefoot region. Moreover, the types of patient were varied in terms of risk level for ulceration with none of the previous studies specifically investigating patients with a confirmed history of foot ulceration. This group merits close attention in order to better understand those at risk from ulceration as they constitute the highest risk of developing a foot ulcer.

Off-loading plantar pressures is a key target in healing and preventing ulceration in diabetes [21]. After healing of a foot ulcer, many patients experience ulcer recurrence yet there is little evidence on risk factors for this event [22,23]. Emerging evidence suggests that barefoot pressures are a significant determinant of ulcer recurrence that has been identified as related to repetitive stress on the foot [10]. As with the first ulcer episode, more knowledge is required on the underlying mechanisms of high barefoot plantar pressure to improve understanding of ulcer recurrence. Furthermore, despite its clinical importance, the measurement of pressure is not widely implemented in clinical practice. But whether plantar pressure and ulcer risk can be predicted from standard clinical measures, or should be directly measured in a high-risk population remains a question of interest. Therefore the aims of this study were to determine which factors can predict barefoot dynamic plantar pressure in an at-risk population with diabetic neuropathy and a history of ulceration, and to establish recommendations for foot screening and management in this high-risk group.

## Methods

### Patients

Patients with diabetes mellitus, peripheral neuropathy, and a recent history of plantar foot ulceration were eligible for inclusion. The study is a cross sectional study and a sub analysis of data collected in the DIAbetic Foot Orthopedic Shoe (DIAFOS) trial [22]. Exclusion criteria were inability to walk 100m unaided and bilateral amputation proximal to the metatarsals.

### Ethics Statement

Ethical approval was obtained from the medical ethics committee of the Academic Medical Centre, University of Amsterdam and all participants provided written informed consent prior to study entry.

### Demographic, disease and foot assessment

Demographic information was recorded at study entry including: age, gender, duration of diabetes, glycosylated haemoglobin levels and body mass. Foot deformity was recorded as present or absent with regard to the following: Charcot midfoot deformity, pes planus, pes cavus, hammer toes, claw toes, hallux abducto valgus and amputation (i.e. digit, ray, or forefoot). The scoring of deformity was undertaken during physical examination by one of three trained investigators and confirmed by two teams of two trained observers who scored standardised images of the feet and reached consensus on outcome. The presence of midfoot deformity based on Charcot neuro-osteoarthropathy was additionally verified from the medical records of affected patients. Hallux abducto valgus was defined as lateral deviation of the hallux relative to the first metatarsal, hammer toes as hyperflexion at the proximal interphalangeal joints with corresponding apical ground contact and claw toes as hyperextension at the metatarso-phalangeal (MTP) joints with hyperflexion at the interphalangeal joints of the lesser toes. Pes cavus was defined as a high medial-longitudinal arch and pes planus as a lowered medial-longitudinal arch; both were assessed weight-bearing. Presence of abundant callus at study entry and prior ulceration specific to each region were recorded dichotomously.

Prominent metatarsal heads, defined as palpable bony prominences, were diagnosed based on physical assessment by one of the three trained investigators. Ankle joint range of motion (ROM) was recorded via goniometry in the supine position. The range of dorsiflexion of the hallux was recorded relative to the first metatarsal shaft; bisection lines were drawn medially along the shaft of the first metatarsal and the proximal phalanx of the hallux and measured with a goniometer in the supine position [24]. Weight-bearing dorsiflexion of the hallux was recorded as the maximal angle of dorsiflexion passively achieved relative to the weight-bearing surface. All goniometric measurements were recorded twice and the mean was entered into the analysis. Peripheral neuropathy was confirmed present in each patient by the inability to sense the pressure of a 10 gram Semmes-Weinstein monofilament at minimum one of three locations on the plantar foot or by a vibration perception threshold at the dorsal aspect of the hallux greater than 25 Volts recorded using a Bio-Thesiometer (Biomedical Instrument Company, Newbury, OH, USA)[2].

### Plantar pressure analysis

Barefoot plantar pressures during normal walking were recorded using an EMED-X (Novel GmbH, Munich, Germany) pressure platform using the two-step method [25] from four walking trials. The platform has a spatial resolution of four sensors per cm^2^ and was sampled at 70Hz. Pressures were analysed using Novel multimask software (version 13.3.65) in five distinct regions of the foot based on functional regions and areas susceptible to local deformity: the heel, midfoot, forefoot (i.e. metatarsals), lesser toes and hallux. The mean peak pressure from the four trials in each of the regions was used in the analysis as the outcome variable.

### Potential predictor variables

Only variables with a realistic potential contribution to the outcome variable based on indications from the scientific literature were included. Therefore, for example, forefoot deformities were not considered relevant to the heel model [26]. Potential predictor variables included: age, gender, body mass, duration of diabetes, glycosylated haemoglobin levels, vibration perception threshold, presence of abundant callus, ankle joint ROM, hallux dorsiflexion range of dorsiflexion, and the following foot deformities: Charcot midfoot deformity, pes planus, pes cavus, hammer toes, claw toes, prominent metatarsal heads, hallux abducto valgus, and amputation.

### Statistical analysis

Statistical analyses were performed using SPSS 20.0 (SPSS Inc., Chicago, IL, USA). Demographic and group characteristics were summarised with the mean (standard deviation, [SD]), median (interquartile range) or number of cases. Differences in peak plantar pressures between presence and absence of dichotomous predictor variables (i.e. all deformities where appropriate, gender, prior ulceration and presence of abundant callus) were assessed for each foot region using Mann-Whitney U tests. Relationships were explored between peak plantar pressures in each region and continuous predictor variables (body mass, age, diabetes duration, vibration perception threshold, ankle and hallux dorsiflexion ROM and glycosylated haemoglobin) using Spearman’s correlation. Data were pooled for left and right limbs to increase statistical power and avoid missing relevant feet. The majority of independent predictors entered into the model are at the foot rather than the person level, therefore the anticipated interdependency between limbs is low.

Pressure variables with skewed data were log transformed. Univariate regression analyses were used to explore the relationship of each potential predictor variable with peak plantar pressure in each of the five regions of the foot. Significant factors with a value of P<0.20 were included in the multivariable linear regression model with backward selection and considered significant with P<0.05. All results were checked with regard to assumptions of multivariate regression analysis including multicollinearity, normality, homoscedascity and independence of residuals.

## Results

### Group characteristics

171 patients (141 male, 30 female) were recruited from 10 Dutch hospitals between January 2008 and October 2010. Four participants were unable to perform barefoot pressure measurement and five participants had below knee or foot amputation affecting one limb and were unable to provide barefoot plantar pressures on one side. Therefore, a total of four participants and an additional five feet were excluded resulting in 167 participants (138 male, 29 female) contributing 329 feet to the barefoot pressure analysis. The included participants had a mean (SD) age of 63 (10) years with mean (SD) duration of diabetes of 17 (13) years. A variety of foot deformities were present with hammer toes being the most prevalent deformity. Twenty-two feet had Charcot midfoot deformity and amputations ranged from partial digital to transmetatarsal. Prior ulceration occurred across all foot regions; only one case occurred in the heel, therefore this variable was not included in the regression analyses for the heel region. Results for demographic and disease related predictor variables are summarised in Table 1.

**Table 1:** Summary of all potential predictor variables in 167 analysed patients.

Variable	Mean (SD)/ Median [IQR] or N
Gender (male/female)	138/29
Age (years)	63 (10)
Body mass (kg)	99 (21)
Duration of diabetes (years)	17 (13)
Glycosylated haemoglobin (%)	7.6 (1.4)
Vibration perception threshold (volts)	50 [40, 50]
Hallux dorsiflexion ROM, non-WB (degrees)	65 (22)
Hallux dorsiflexion ROM, WB (degrees)	21 (14)
Ankle joint ROM (degrees)	51 (17)
Hallux abducto valgus (present/absent)	76/224
Claw toes (present/absent)	79/241
Hammer toes (present/absent)	94/227
Prominent metatarsal heads (present/absent)	87/242
Charcot midfoot deformity (present/absent)	22/307
Pes planus (present/absent)	73/255
Pes cavus (present/absent)	32/296
Abundant callus (present/absent)	77/252
Partial foot amputation (yes/no), of which:	67/262
Digital/partial digital, including hallux	28
Metatarsal head	4
Ray (toe + metatarsal)	19
Transmetatarsal	16
Previous foot ulceration (yes/no), of which at:	166/163
Heel	1
Midfoot	11
Forefoot	81
Lesser toes	33
Hallux	40

Key: ROM–range of motion, WB–weightbearing.

### Plantar pressure features

Barefoot peak pressures recorded were highest in the forefoot region, median (IQR) 830kPa (526, 1112), and lowest in the midfoot region, 141kPa (94, 214) for the entire cohort. Plantar pressure data were not normally distributed in three of the five regions (heel, midfoot and lesser toes) and were log transformed prior to regression analyses. A descriptive summary of all outcome variables is presented for the entire cohort and also grouped by categorical predictor variable for each region of the foot in Table 2. Feet may have presented with multiple deformities, therefore absence of a single deformity in Table 2 does not equate to a foot without deformity. The association of the continuous predictor variables to plantar pressure in each region is presented in Table 3. Due to the distribution characteristics of some of the variables, for consistency all data are presented as median (inter-quartile range) and results are from non-parametric tests.

**Table 2:** Descriptive summary of barefoot plantar pressure by categorical predictor variables.

Median (interquartile range) peak pressure by region					
Variable	Heel	Midfoot	Forefoot	Lesser toes	Hallux
**All subjects**	330 (259, 406)	141 (94, 214)	830 (526, 1112)	212 (112, 345)	391 (226, 717)
**Prev. ulcer**					
Present	N/A	347 (250, 1275)	1030 (806, 1192)	305 (223, 478)	791 (433, 1132)
Absent	N/A	138 (93, 206)	873 (608, 1144)	200 (100, 334)	376 (214, 640)
P-value	N/A	0.000**	0.002**	0.000**	0.000**
**Callus**					
Present	329 (261, 403)	149 (104, 215)	1024 (806, 1215)	219 (101, 332)	460 (233, 846)
Absent	331 (258, 409)	139 (93, 215)	750 (483, 1067)	212 (116, 345)	386 (221, 690)
P-value	0.998	0.542	0.000**	0.625	0.078
**Gender**					
Male	330 (267, 400)	141 (95, 210)	804 (506, 1132)	195 (99, 350)	392 (228, 721)
Female	334 (201, 502)	134 (85, 284)	911 (590, 1076)	248 (177, 331)	389 (219, 690)
P-value	0.993	0.775	0.509	0.096	0.800
**HAV**					
Present	315 (242, 380)	162 (126, 234)	1015 (682, 1172)	230 (101, 345)	438 (205, 733)
Absent	334 (270, 421)	128 (85, 189)	726 (476, 1021)	208 (114, 345)	388 (239, 712)
P-value	0.088	0.001**	0.000**	0.829	0.738
**Claw toes**					
Present	315 (228, 382)	153 (98, 229)	1041 (824, 1200)	190 (68, 344)	359 (152, 701)
Absent	333 (269, 420)	136 (91, 207)	716 (476, 1030)	229 (120, 347)	399 (248, 716)
P-value	0.108	0.119	0.000**	0.097	0.209
**Hammer toes**					
Present	330 (270, 404)	156 (117, 290)	824 (502, 1142)	288 (199, 499)	395 (222, 743)
Absent	332 (258, 408)	134 (85, 204)	825 (528, 1098)	178 (95, 290)	389 (227, 712)
P-value	0.999	0.002**	0.899	0.000**	0.873
**Prom. MTH**					
Present	348 (278, 449)	136 (90, 209)	1069 (908, 1247)	204 (70, 290)	308 (109, 607)
Absent	321 (253, 391)	142 (95, 217)	710 (467, 1016)	230 (121, 348)	432 (259, 739)
P-value	0.030*	0.395	0.000**	0.065	0.001**
**Charcot**					
Present	299 (259, 399)	756 (234, 1274)	951 (444, 1198)	183 (76, 381)	241 (40, 626)
Absent	330 (258, 408)	137 (93, 197)	825 (527, 1110)	221 (114, 344)	399 (241, 720)
P-value	0.513	0.000**	0.647	0.530	0.045*
**Pes planus**					
Present	283 (216, 381)	185 (136, 316)	916 (492, 1164)	233 (136, 389)	540 (367, 780)
Absent	336 (275, 411)	131 (86, 186)	822 (531, 1070)	200 (105, 344)	370 (195, 692)
P-value	0.002**	0.000**	0.483	0.253	0.002**
**Pes cavus**					
Present	377 (319, 468)	145 (90, 185)	916 (581, 1226)	141 (88, 286)	220 (125, 438)
Absent	327 (258, 403)	140 (94, 222)	824 (518, 1098)	223 (120, 348)	413 (249, 736)
P-value	0.038*	0.515	0.367	0.057	0.001**
**Amputation**					
Yes	306 (219, 396)	157 (118, 263)	1113 (808, 1242)	219 (68, 298)	456 (310, 1066)
No	333 (265, 410)	138 (90, 207)	763 (504, 1034)	208 (115, 348)	388 (220, 711)
P-value	0.178	0.026*	0.000**	0.426	0.312

Key: P-value from Mann-Whitney U test comparing presence and absence of categorical parameter, ** <0.005, * <0.05, pressure values presented in kPa, Prev. ulcer- previous ulceration, HAV–hallux abducto valgus, Prom. MTH–prominent metatarsal heads. Feet may have presented with multiple deformities, therefore absence of a single deformity does not equate to a foot without deformity.

**Table 3:** Summary of correlations between barefoot plantar peak pressure and continuous predictor variables.

Variable	Heel	Midfoot	Forefoot	Lesser toes	Hallux
Body mass	.066 (0.236)	.358 (0.000)**	.024 (0.666)	.016 (0.771)	.063 (0.276)
Age	-.124 (0.025)*	-.083 (0.135)	.163 (0.003)**	.125 (0.025)*	.031 (0.593)
Duration diabetes	.123 (0.026)*	-.106 (0.057)	-.019 (0.733)	-.045 (0.427)	.022 (0.700)
Vibration perception threshold	.041 (0.460)	.001 (0.979)	.045 (0.419)	.009 (0.879)	.007 (0.905)
Ankle ROM	.107 (0.056)	-.289 (0.000)**	-.223 (0.000)**	.108 (0.056)	.004 (0.947)
Hallux dorsiflexion ROM, non-WB	.047 (0.419)	-.014 (0.809)	.106 (0.069)	-.162 (0.005)**	-.199 (0.001)**
Hallux dorsiflexion ROM, WB	-.086 (0.140)	.023 (0.693)	-.046 (0.435)	-.086 (0.140)	-.175 (0.003)**
Glycosylated haemoglobin	.212 (0.000)**	-.050 (0.382)	-.066 (0.250)	-.106 (0.064)	.041 (0.488)

Key: Spearman’s correlation, P-value in brackets, *<0.05, **<0.005, ROM–range of motion, WB—weightbearing.

### Regression analyses

In the univariate analyses, all factors, with the exception of vibration perception threshold, were associated (P <0.20) with peak plantar pressure in at least one of the five studied regions of the foot and were therefore entered into at least one of the multivariate models. In the multivariate regression analyses for each region the following factors were significantly independently associated with peak plantar pressure (P<0.05): glycosylated haemoglobin and pes planus in the heel; Charcot midfoot deformity, body mass, ankle joint ROM, previous ulceration and pes planus in the midfoot; prominent metatarsal heads, claw toes, abundant callus, amputation, ankle joint ROM, previous ulceration and age in the forefoot; hammer toes, non-weight bearing hallux dorsiflexion ROM, and previous ulceration in the lesser toes; prominent metatarsal heads, pes cavus, pes planus, weight bearing hallux dorsiflexion ROM, abundant callus and previous ulceration in the hallux region. The multivariate models were capable of explaining between 6% and 41% of the variance in barefoot plantar pressure. The univariate and multivariate regression results are summarised in Table 4; the standardised beta weights are presented which represent the relative contribution of each variable to the explanation of variance in plantar pressure.

**Table 4:** Univariate and multivariate regression analyses.

Model R^2^ (P-value)	Independent variables	Univariate standardised Beta (P-value)	Multivariate standardised Beta (P-value)
**Heel**	Ankle joint ROM	.123 (0.027)	.095 (0.096)
0.063	Glycosylated haemoglobin	.178 (0.002)	.185 (0.001*)
(0.000*)	Pes planus	-.146 (0.008)	-.127 (0.027*)
**Midfoot**	Body mass	.327 (0.000)	.198 (0.000*)
0.413	Duration of diabetes	-.119 (0.032)	
(0.000*)	Ankle joint ROM	-.315 (0.000)	-.148 (0.001*)
	Charcot midfoot deformity	.411 (0.000)	.504 (0.000*)
	Pes planus	.223 (0.000)	.117 (0.010*)
	Amputation	.131 (0.018)	
	Previous midfoot ulceration	.345 (0.000)	.095 (0.049*)
**Forefoot**	Age	.176 (0.001)	.129 (0.008*)
0.311	Ankle joint ROM	-.182 (0.001)	-.102 (0.038*)
(0.000*)	Hallux dorsiflexion ROM, non-WB	.099 (0.090)	
	Glycosylated haemoglobin	-.106 (0.062)	
	Abundant callus	.254 (0.000)	.177 (0.000*)
	Hallux abductor valgus	.212 (0.000)	
	Claw toes	.317 (0.000)	.219 (0.000*)
	Prominent metatarsal heads	.373 (0.000)	.252 (0.000*)
	Amputation	.274 (0.000)	.121 (0.017*)
	Previous forefoot ulceration	.173 (0.002)	.118 (0.020*)
**Lesser**	Age	.115 (0.042)	
**toes**	Duration diabetes	-.078 (0.174)	
0.174	Ankle joint ROM	.102 (0.076)	
(0.000>*)	Hallux dorsiflexion ROM, non-WB	-.203 (0.001)	-.112 (0.040*)
	Glycosylated haemoglobin	-.135 (0.021)	
	Gender	.085 (0.133)	
	Claw toes	-.091 (0.109)	
	Hammer toes	.319 (0.000)	.332 (0.000*)
	Prominent metatarsal heads	-.093 (0.100)	
	Pes cavus	-.121 (0.033)	
	Previous lesser toe ulceration	.199 (0.000)	.158 (0.004*)
**Hallux**	Hallux dorsiflexion ROM, non-WB	-.184 (0.002)	
0.208	Hallux dorsiflexion ROM, WB	-.157 (0.008)	-.138 (0.011*)
(0.000*)	Abundant callus	.126 (0.029)	.119 (0.028*)
	Prominent metatarsal heads	-.162 (0.005)	-.143 (0.009*)
	Charcot midfoot deformity	-.086 (0.137)	
	Pes planus	.152 (0.008)	.093 (0.090)
	Pes cavus	-.171 (0.003)	-.167 (0.002*)
	Previous hallux ulceration	.329 (0.000)	.321 (0.000*)

Key: ROM–range of motion, WB–weightbearing, * P<0.05 in multivariate model.

## Discussion

The aims of this study were to determine which factors could predict barefoot plantar pressures in a high-risk population with diabetic neuropathy and a history of ulceration and to establish recommendations for foot screening and management in this high-risk group. The predictor variables were capable of explaining between 6% and 41% of the variance in peak pressures in the multivariate regression analyses in the five different foot regions. In the midfoot region the most variation in plantar pressures (41%) was explained of all foot regions, with the most significant contribution from the presence of Charcot midfoot deformity. Furthermore, Charcot deformity showed the highest predictor value (Beta coefficient 0.504) of any factor in any of the foot regions studied. In the forefoot region, 31% of variation in pressure was explained with the largest contribution from the presence of prominent metatarsal heads, followed by claw toes. These factors were expected and confirm findings from previous studies with regard to the role of foot deformity in plantar pressure in diabetic patients [12,15,18,19]. In the majority of the five models, local factors such as the presence of foot deformity or a prior foot ulcer were clearly stronger predictors of plantar pressure than global features (age, gender, body mass, duration of diabetes, or vibration perception threshold). Therefore, deformity should be adequately managed in clinical practice to reduce plantar pressure and ulcer risk. Furthermore, the data stress and confirm that the region where the previous foot ulcer was present, remains an important target for pressure relief. However, a large amount of variance in barefoot plantar peak pressure remains unexplained in this high-risk population with diabetes. This suggests that measurement of plantar pressure as a ‘surrogate’ of foot injury should be an integral part of foot screening for these high-risk patients.

Few global factors were significantly associated with pressure in the multivariate models: age in the forefoot model, body mass in the midfoot model and HbA1c in the heel model. Age related changes to stiffness of the plantar soft tissues [27] and reduction in joint motion [28] have been reported and may impact upon forefoot plantar pressure. The general lack of significant relationship found between body mass and plantar pressures is in agreement with previously reported research [14,18]. In one previous study, body mass index showed no significant relationship with peak plantar pressure in the forefoot whereas soft tissue thickness demonstrated a significant inverse association with plantar pressures [20]. The authors postulated this was perhaps a result of those with higher body mass having more subcutaneous tissue [20]. In the current study, body mass remained a significant predictor only in the midfoot model and it did not explain the largest amount of variation relative to the other predictor variables. Contrary to commonly held beliefs, there is little data to support the role of body mass in determining barefoot dynamic plantar pressures in patients with diabetes.

Ankle joint ROM emerged as a significant predictor variable in three of the five models (all regions except lesser toes and hallux). Limited joint mobility in diabetes, also known as diabetic cheiroarthopathy, is associated with collagen glycosylation resulting in proliferation of peri-articular tissue [29]. Reduced ankle joint ROM has been reported in patients with diabetes and has been linked to increased plantar pressures [30] and plantar ulceration [31]. In further support of this association, Achilles tendon lengthening procedures have been shown to be effective in increasing ankle joint dorsiflexion, reducing forefoot plantar pressures and reducing forefoot neuropathic ulcer recurrence [32].

Hallux dorsiflexion ROM was a significant predictor in the hallux and lesser toe models. This confirms previous observations of an association between either static or dynamic hallux ROM and plantar pressure [13,16,33]. Static joint ROM measurements have been reported to have limited ability to predict dynamic joint angular movements [34]. However, a correlation has been reported between passive and active motion at the hallux in patients with diabetes, together with a positive association with peak forefoot pressures [35]. The present study additionally recorded weight-bearing dorsiflexion ROM, a commonly used clinical technique to diagnose functional hallux limitus. Only weight bearing hallux dorsiflexion remained significant in the hallux model suggesting that it should continue to be measured in clinical practice. However, it should be borne in mind that the hallux model explained only 13% of variance in plantar pressures in the studied cohort.

With regard to the lesser-toe pressures, the largest single contribution came from hammer toe deformity which is in agreement with previous research [15]. Interesting in this regard is that toe flexor muscle tendon tenotomy procedures have been successfully employed to heal and prevent apical toe ulcers in patients with diabetes, with post-surgical pressure reduction the most likely mechanism [36]. Hallux abducto valgus was not a significant predictor in the hallux or lesser toe models but this is perhaps related to the fact the deformity predominantly affects the transverse and frontal planes rather than the sagittal.

The models were capable of explaining only between 6% and 41% of variance in barefoot plantar pressures in the studied cohort. Adding factors that were not recorded in this study on clinical correlates may improve predictive value. Data on dynamic gait, such as kinematics and kinetics, is one such factor [37]. Another dynamic variable of interest is walking speed, which has been shown to mediate plantar pressure in the heel and forefoot regions [38]. A study in a non-diabetic population combined both structural and functional factors and was able to predict between 49–57% of variance in plantar pressures, even though outcomes varied considerably across foot regions and structural variables were shown to be more contributory than functional factors [33]. Finally, foot muscle strength and morphology are affected by diabetes and neuropathy and may influence plantar pressure [39]. While adding these factors may improve prediction of plantar pressure, none of these variables are measured in a standard clinical setting and may therefore have limited clinical applicability.

This study was subject to limitations: first, the study was part of a larger trial and pragmatically it was not possible to collect all potential variables that may be of interest in explaining foot pressure. The main interest was to investigate whether prediction of barefoot pressures was possible from standard clinical measures. Secondly, the presence of multiple deformities in the same foot was not controlled for and may have been a potential limitation of the study in contributing to a lower explained variance in the models. Finally, the study focused on a high-risk population, which by virtue of the inclusion criteria may have resulted in masking the importance of certain variables. For example, VPT was not a strong predictor in any of the models, likely because participants who entered the study were all neuropathic. However, the inclusion of only a high-risk sample population was also a key strength of this study due to the morbidity associated with foot ulceration and the high ulcer recurrence rates in this group. Therefore, any new insights add to our current understanding of this high-risk population. Furthermore, this sample of high-risk patients was recruited from ten academic or large community-based Dutch hospitals and therefore the results are generalizable to the high-risk diabetic population.

In conclusion, this study has demonstrated poor to moderate prediction of barefoot plantar pressures in diabetic patients who are at highest risk of developing plantar foot ulcers, those with neuropathy and a history of plantar ulceration. Local factors (such as deformity) were better predictors than global features (such as age or body mass) and deformity should therefore be adequately managed to reduce plantar pressure and ulcer risk. While it is acknowledged that no clear data yet indicate that the screening for barefoot plantar pressures can help to reduce the incidence of foot ulceration, the study results suggest that measurement of plantar pressure as a ‘surrogate’ of foot injury should be an integral part of foot screening as no single factor in this study has emerged as an adequate proxy measure.
